# Enhancement of Hydrotropic Fractionation of Poplar Wood Using Autohydrolysis and Disk Refining Pretreatment: Morphology and Overall Chemical Characterization

**DOI:** 10.3390/polym11040685

**Published:** 2019-04-15

**Authors:** Yanting Gu, Huiyang Bian, Liqing Wei, Ruibin Wang

**Affiliations:** 1 Jiangsu Co-Innovation Center of Efficient Processing and Utilization of Forest Resources, Nanjing Forestry University, Nanjing 210037, China; hybian1992@njfu.edu.cn; 2 College of Furnishings and Industrial Design, Nanjing Forestry University, Nanjing 210037, China; 3 Jiang Provincial Key Lab of Pulp and Paper Science and Technology, Nanjing Forestry University, Nanjing 210037, China; 4 Forest Products Laboratory, U.S. Forest Service, U.S. Department of Agriculture, Madison, WI 53726, USA; liqingwei1325@gmail.com; 5 School of Materials and Energy, Center of Emerging Material and Technology, Guangdong University of Technology, Guangzhou 510006, China; wang.rb@gdut.edu.cn

**Keywords:** hydrotropic fractionation, autohydrolysis, disk refining, fiber morphology, chemical composition

## Abstract

Solid acids have been proposed as a hydrolytic agent for wood biomass dissolution. In this work, we presented an environmentally friendly physicochemical treatment to leave behind cellulose, dissolve hemicellulose, and remove lignin from poplar wood. Several pretreatments, such as autohydrolysis and disk refining, were compared to optimize and modify the process. The *p*-toluenesulfonic acid could extract lignin from wood with a small amount of cellulose degradation. Disk refining with subsequent acid hydrolysis (so-called physicochemical treatment) doubled the delignification efficiency. A comprehensive morphology and overall chemical composition were provided. The crystallinity index (CrI) of treated poplar was increased and the chemical structure was changed after physicochemical treatment. Optical microscopy and scanning electron microscopy analysis demonstrated physicochemical treatment affected the morphology of poplar wood by removing lignin and generating fiberization. In general, this work demonstrated this physicochemical method could be a promising fractionation technology for lignocellulosic biomass due to its advantages, such as good selectivity, in removing lignin while preserving cellulose.

## 1. Introduction

The industrial utilization of lignocellulosic biomass has been considered as the key for access to an integrated production of chemicals, materials and energy of the future. Lignocellulosic materials (LCMs) may potentially replace petroleum as a raw material to obtain a variety of products adapted to market demands [1]. The next generation of biofuel will be produced from lignocellulosic feedstocks (wood, agricultural residues, and dedicated energy crops) through the fermentation of sugars yielded from hydrolysis of two key components of lignocellulose: the cellulose and hemicellulose, but lignin, as the abundant biopolymer, is underutilized [2,3].

Wood biomass is a very attractive form of renewable feedstock due to its low cost of production, availability in large quantities and easy storage. However, major barriers must be overcome for the efficient conversion of woody lignocellulose to a simple component. Extensive research efforts have been devoted to various pretreatments of biomass to overcome the physical and chemical resistance of plant cell structure. This resistance is often called recalcitrance, which can be reduced by partially removing hemicellulose and lignin. Hemicelluloses with low molecular mass are amorphous and branched, and in this form, they are easily soluble in water, however, less branched hemicelluloses are tightly bound to celluloses and less soluble in water [4]. Removal of hemicelluloses in a pure form needs hydrolysis of covalent bonds linking the hemicellulose to lignin. In the biorefinery concept, prehydrolysis (autohydrolysis), based on the utilization of hot, compressed water as a fraction agent, has been utilized to allow the selective separation of major parts of hemicelluloses in the form of oligosaccharides, sugar, acetic and formic acids [5]. Lignin, the most abundant natural aromatic polymer, has a highly branched three-dimensional phenolic structure. Most lignin is a by-product derived from the paper pulping process, so lignin has long been given the label of a “waste material”. To obtain a good efficiency of bioconversion, lignin as the main barrier needs to be removed using the kraft process, hydrotropic pretreatment and bleaching method [6,7,8].

Wood is known to be recalcitrant towards chemical conversion. Thereby, for high value products production, such as dissolving pulp with a high cellulose content and a minimum amount of non-cellulosic impurities, hemicelluloses dissolution and delignification are a tremendous challenge for so-called integrated forest products biorefinery concepts [9]. Until now, many published studies have been focused on the dissolution of wood biomass. Acid sulfite and vapor-phase prehydrolysis kraft (PHK) processes were both developed to dissolve wood pulps in the 1950s [6]. Wood also could be dissolved and chemically modified from chloride ionic liquids, but the degree of degradation caused during the process was not apparent [10]. A better solubilisation of wood and more efficient fractionation was discovered after initial autohydrolysis pretreatment with subsequent ionic liquid fractionation [6]. In the paper pulping industry, autohydrolysis caused a significant increase in the delignification rate during subsequent kraft or soda-anthraquinone (SAQ) pulping [11]. The required degree of delignification can be achieved by controlling the autohydrolysis condition. However, alkaline delignification is related to high cellulose losses due to the β-elimination even under mild conditions. In order to preserve the cellulose yield during the delignification process, hydrotropic treatment with a concentrated aqueous solution of agents, such as sodium xylene sulfonate (SXS), was introduced for delignification. Recently, hydrotropic pretreatment was investigated to remove more lignin from birch wood compared to the hydrothermal treatment and room temperature ionic liquid method [12]. In addition, to retain large amounts of cellulose, the hydrotropic lignin can be recovered from the spent solution. Solid acids, such as oxalic acid and maleic acid, also have been used to hydrolyze cellulose for sugar with the advantage of easing acid recovery [13,14]. However, using solid acid to separate lignin for fractionation of wood biomass to obtain high-valued pulp is still not reported.

Herein, we propose a separation of lignin from poplar wood using an aromatic acid, p-toluenesulfonic acid (p-TsOH) at a low temperature in a short time. This acid has been used for enzymatic saccharification [15], furfural production [16], agricultural waste utilization [17] and lignocellulose nanomaterials production [18]. This acid was found to have capability for the rapid and nearly-complete dissolution of wood lignin below the boiling temperature of water. It also can be relatively easily recycled and maintain cellulose and hemicellulose for a low energy input. Further, the *p*-TsOH solubilized lignin can be recovered as lignin nanoparticles that form valuable building blocks. And the effect of different pretreatments, autohydrolysis and disk refining, on the efficiency of fractionation of wood was also investigated. The main aim of this research was to compare and modify the treatment to strengthen lignin separation and obtain pulp with high cellulose content.

## 2. Materials and Methods 

### 2.1. Materials

The *p*-toluenesulfonic acid (*p*-TsOH) was ACS reagent grade and purchased from Sigma-Aldrich (St. Louis, MO, USA). Wood logs of poplar NE222 (Populus deltoides Bartr. ex Marsh×Populus nigra L.) was chipped using a knife chipper. The wood chips were screened to remove the majority of materials larger than 38 mm and less than 6 mm in length. The thicknesses of the chips ranged from 1 to 5 mm. The chips were kept frozen at −16 °C until use.

### 2.2. Autohydrolysis

The experiment was prepared by extracting the poplar wood with hot water. In this study, autohydrolysis was conducted in 1 L pressure vessels and each run used 30 g wood chips (in oven dry weight), together with a predetermined amount of deionized water to reach a liquid-to-wood (L:W) ratio of 4:1 or total solid loading of 25 wt %. Three 1 L pressure vessels were mounted into a 23 L rotating digester heated by a steam jacket in an autoclave configuration as described previously [19]. The digester was rotated end-for-end at 2 rpm for mixing. Autohydrolysis was conducted at 170 °C for approximately 50 min. At the end of the reaction, the digester was cooled by flushing cold tap water into the jacket before opening and three reactors inside were further cooled. The remaining wood residues were washed several times using deionized water and then stored in plastic bags for further processing.

### 2.3. Wood Size Reduction

Untreated and autohydrolyzed poplar wood were directly disk refined in a 12-inch laboratory disk refiner (Andritz Sprout-Bauer Atomospheric Refiner, Springfield, OH, USA) using two-disks with plate pattern DB2-505 at a disk plate gap of 1 mm, approximately 10 times larger than that used for typical mechanical pulping. The energy consumption for milling was minimal due to the large plate gap used.

### 2.4. Hydrotropic Fractionation

**Figure 1:**
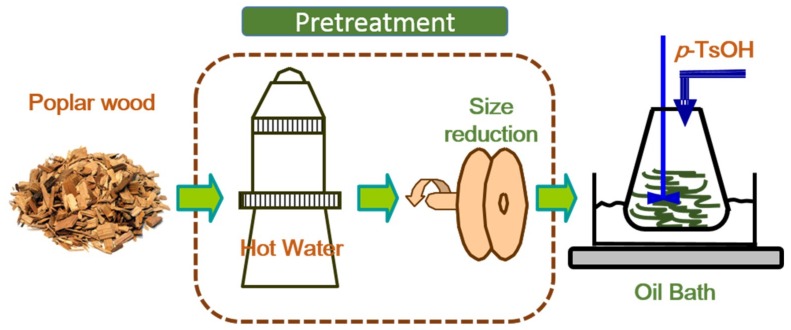
A schematic experimental flow diagram for enhancement of hydrotropic fractionation of poplar wood using autohydrolysis and disk refining pretreatment.

Delignification experiments were conducted in a liquid glycerol bath on a heating plate with the assistance of magnetic stirring. The required amounts of *p*-TsOH and deionized (DI) water were added in a 500 ml three-necked flask to make a desired concentrated acid solution. When the solution was heated to the desired hydrolysis temperature, oven dry (OD) weight samples were fed continuously into the completely dissolved acid solution with a liquor to wood chips ratio of 10:1. A total of 12 experiments were conducted in a range of reaction conditions. Reactions were conducted at temperatures of 75 and 80 °C with an acid concentration range of 70–80 wt % in 5 wt % increment. Acid hydrolysis was terminated at the end of the predetermined reaction time of 20 min. The mixture was filtered by vacuum filtration using a filter paper (15cm, slow, Fisher Scientific Inc., Pittsburgh, PA, USA). Hydrolyzed solid residues were washed using DI-water until the conductivity of the liquid approached that of DI-water, indicating near-complete acid removal. The washed solids were collected for yield determination using gravimetric method. The approach used in this work is schematically presented in Figure 1.

### 2.5. Characterization

Samples were oven dried at 105 °C overnight, then cooled down and Wiley milled to 20 mesh (model No. 2, Arthur Thomas Co., Philadelphia, PA, USA). The milled samples were hydrolyzed using sulfuric acid in two steps for carbohydrates and lignin analyses as described previously [3]. The effectiveness of pretreatments was evaluated by solid yield (Y_solid_), remaining rate of glucan, xylan and lignin (R_glucan_. R_xylan_ and R_lignin_). The calculation equations were listed as follows:

(1) $$Y_{\mathrm{solid}}\left(\%\right)=\frac{\mathrm{Residual}\;\mathrm{solids}\;\left(g\right)}{\mathrm{Original}\;\mathrm{materials}\;\left(g\right)}\times 100$$

(2) $$R_{z}=\frac{Y_{\mathrm{solid}}\times C_{z\;\mathrm{of}\;\mathrm{the}\;\mathrm{residual}\;\mathrm{solids}}}{C_{z\;\mathrm{of}\;\mathrm{the}\;\mathrm{original}\;\mathrm{material}}}$$

where C_z_ is the percentage of the z (glucan, xylan and lignin) in the corresponding material.

Radial and longitudinal section of untreated and *p*-TsOH treated poplar wood blocks (20 µm thick) were obtained with a Reichert microtome (Reuchert, Vienna, Austria) at room temperature. The sections were transferred to glass slides. Staining with 1% aqueous Safranin O (Sigma catalog no. S2255) was carried out for 3 to 5 min without removing them, then covered with coverslips. The section was rinsed with water after each staining step and then embedded in glycerol and examined under the Eclipse Ci microscope (Nikon, Tokyo, Japan) equipped with a Flea 3 camera. Images of untreated and *p*-TsOH treated poplar wood were also observed using scanning electron microscopy (SEM, Carl Zeiss NTS, Peabody, MA, USA). Before observation, the samples were prepared drying a small amount of suspension on a well-polished aluminum mount and sputter-coating with a gold layer.

The crystallinity of raw material and treated poplar was analyzed using wide-angle X-ray diffraction on an X-ray diffractometer under a Bruker D8 130 Discover system with Cu-Kα radiation (Bruker Corp., Billerica, MA, USA). Samples were pressed at 200 MPa to make pellets as described previously [20]. The scattering angle (2θ) ranged from 10° to 38° in steps of 0.02°. The crystallinity index (CrI) was calculated according to the Segal method (without base line subtraction) [21].

The chemical structure of the samples was recorded using a commercial FTIR spectrophotometer with a universal attenuated-total-reflection (ATR) probe (Spectrum Two, PerkinElmer, UK). Each sample was analyzed in its dry form. The absorption spectra of the samples were recorded for wavelengths between 450 and 4000 cm^−1^ with a resolution of 4 cm^−1^. Four scans were completed for each sample.

## 3. Results

### 3.1. Chemical Composition of Poplar Wood under Different Treatment Conditions

The chemical composition changes of poplar wood after pretreatments are listed in Table 1. For simplicity, samples were given label designation and abbreviated. As expected, the xylan content was about 7.13 % in the autohydrolyzed (H) wood, only half of the raw material. However, the chemical composition of the *p*-TsOH (P) treated wood was almost the same compared to those subjected to autohydrolysis and *p*-TsOH treatment. This was possibly because poplar chips can be highly recalcitrant to chemical processing depending on their lignin content and structure. Hence, compared with hot water extraction, wood size reduction is critical to increase surface area and accessibility to chemicals or enzymes for biomass conversion. Disk refining (R) before *p*-TsOH hydrolysis was efficient as a delignification aid, which was in agreement with the fact that approximately 40 % lignin was removed after *p*-TsOH treatment (Sample P, R_lignin_ = 0.60), while the delignification was doubled when refining was employed before acid treatment (Sample RP, R_lignin_ = 0.14). In addition, yields of the pulp obtained from disk refining were lower than those without physical pretreatment. Half of the solids were remained after *p*-TsOH hydrolysis, however, approximately 90 % lignin was removed as shown in the Sample HRP and RP, where H, R and P stood for autohydrolysis, disk refining and *p*-toluenesulfonic acid hydrolysis, respectively. Interestingly, glucan loss was very low no matter which pretreatment was employed. This result may be beneficial for the downstream enzymatic saccharification or dissolving pulp production.

### 3.2. Morphology Observation

**Figure 2:**
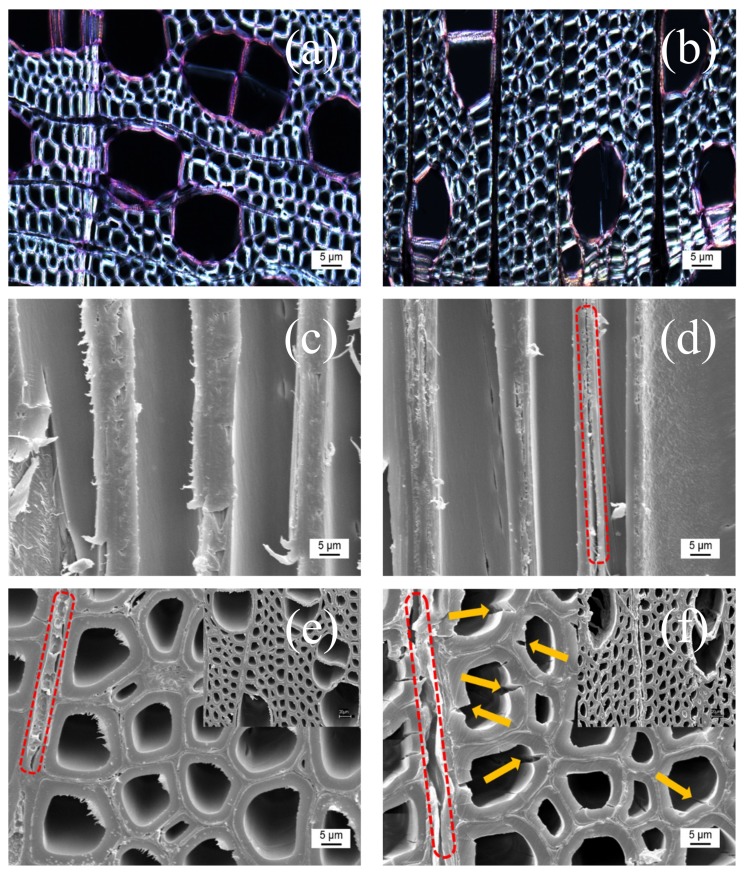
(**a**,**b**) Optical micrographs of the untreated and *p*-TsOH treated poplar wood in radial (R) direction (stained with Safranin O ). (**c**,**d**) SEM images of the untreated and *p*-TsOH treated poplar wood in longitudinal (L) direction, where some cracks were seen in middle lamella (red dashed part). (**e**,**f**) SEM images of the untreated and *p*-TsOH treated poplar wood in radial (R) direction. Some pores or cracks were clearly seen in ray cell after *p*-TsOH treatment (red dashed part). Yellow arrows showed broken cell lumen after *p*-TsOH treatment.

Lignin can be described as a three-dimensional macromolecule originating from phenylpropanoid precursors such as coumaryl, coniferyl, and sinapyl alcohol that are present in vascular plants [22]. It has not been possible to evaluate exactly the degree of delignification; however, the use of stains can prove the presence or absence of lignin in wood tissues. Herein, we used Safranin O to stain lignin regardless of whether cellulose is present in wood samples. Figure 2a–f demonstrated two directions of wood blocks that can be observed from optical microscopy and scanning electron microscopy, where the radially cut piece is named as “R” and the longitudinal one “L”. In R-poplar, the lumen in wood is perpendicular to the plane, while in L-poplar, the lumen is along the plane direction. The color comparison for lignin removal in R-poplar was shown in Figure 2a,b. The reaction of lignin with Safranin O stained it red (less lignified) or pink (high lignified). Since lignin was colored while other components were colorless, the color in R-poplar indicated the amount of lignin present in the radial direction. It was obviously seen that the pink in R-poplar became lighter after *p*-TsOH treatment in Figure 2b, which indicated delignification using *p*-TsOH treatment was effective. We also observed more details about the heterogeneity of delignification patterns and how wood cell walls were attacked by acid. Figure 2b showed the lumen delignified rapidly and the fibers attached to them were often attacked first. This result proved that lignin gradually lost from the lumen towards to middle lamella as the previous work reported [23]. Apart from observation using optical microscopy, SEM also showed the changes of surface structure in R-poplar and L-poplar. The poplar wood block displayed massive lumen along the growth direction. The lumen surrounded by the cell wall was made of cellulose and hemicelluloses in Figure 2c,d. The ray cell (red dashed part) in R-poplar generated some pores and cracks after partial lignin removal. Meanwhile, some broken lumen (yellow arrows) can be clearly seen in Figure 2d due to the loss of lignin. Compared with the untreated poplar in Figure 2e, some cracks were also presented in the middle lamella (red dashed part in Figure 2f) where lignin were enriched. It was astounding that the microstructure with the intact channels was well preserved during the hydrolysis process. Therefore, this physicochemical pretreatment can efficiently remove the lignin while preserving the cellulose from the poplar wood.

**Figure 3:**
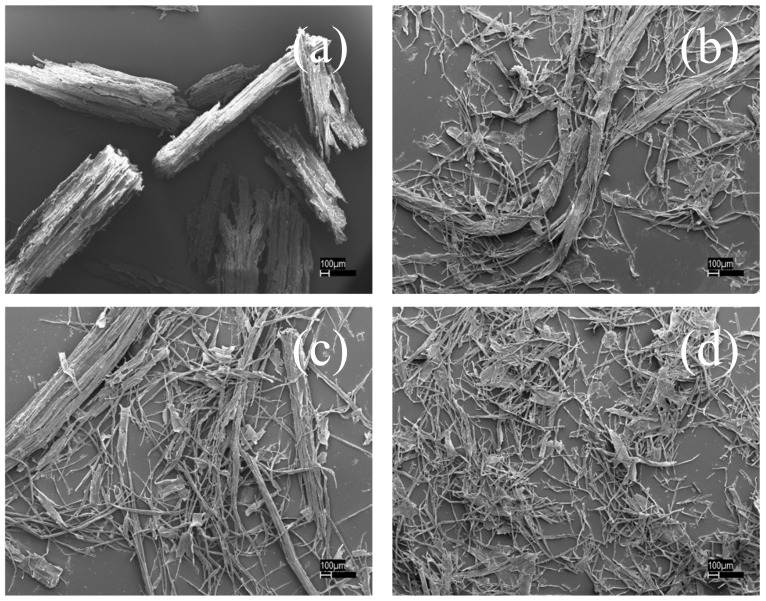
SEM images of poplar wood under different treatment conditions. (**a**) R; (**b**) RP; (**c**) HR and (**d**) HRP. H was autohydrolysis; P was *p*-toluenesulfonic acid hydrolysis; R was disk refining.

Figure 3a,b showed the SEM images of poplar wood under different conditions. Fibers or fiber bundles were all obtained except for the disk-refined poplar in Figure 3a. Autohydrolysis can alter the chemical composition and physical structure of wood by partly removing and modifying some cell-wall components such as hemicellulose, which can reduce energy consumption for refining the autohydrolyzed wood as well as reducing wood size in Figure 3c. Due to the formation of fiberization, *p*-TsOH penetration into wood fiber could be further strengthened. However, the remaining rate of lignin (R_lignin_) was similar, 0.14 for Figure 3b and 0.12 for Figure 3d. As a consequence, wood size reduction was the critical factor for efficient delignification compared with autohydrolysis.

### 3.3. Crystallinity

**Figure 4:**
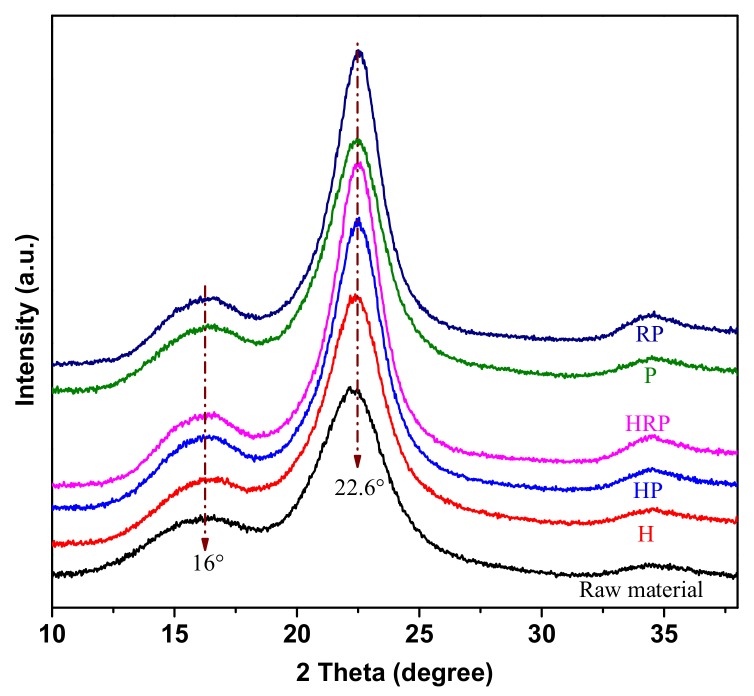
X-ray diffraction patterns of poplar wood under different treatment conditions.

To investigate the influence of different pretreatments on the cellulose crystal structure, the changes in XRD curves of raw and treated poplar were investigated in Figure 4, and the corresponding CrI values were given in Table 1. In all cases, samples exhibited typical diffraction peaks at approximately 16° and 22.6°, which indicated the native celluloseⅠcrystal structure was preserved. Similar results could be found in many published studies [24,25]. The CrI was mainly influenced by the chemical composition. The increase of CrI after pretreatment (particularly RP and HRP) was due to the removal of the amorphous substances, such as lignin and hemicelluloses. In addition, disk refined wood exhibited higher CrI after *p*-TsOH hydrolysis than those samples without size reduction, which was due to the increase in surface area and accessibility to acid after mechanical pretreatment.

### 3.4. Effect of Disk Refining and Hydrotropic Fractionation on the Remaining Rate of Xylan and Lignin

**Figure 5:**
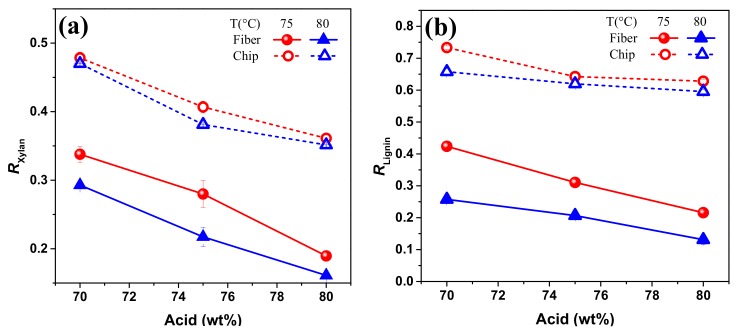
Remaining rate of (**a**) xylan and (**b**) lignin (R_Xylan_ and R_Lignin_) under different concentration of *p*-TsOH hydrolysis. Acid concentration is 70, 75 and 80 wt %; hydrolysis temperature is between 75 and 80 °C; hydrolysis duration is 20 min. Fiber and chip represent poplar with and without disk refining treatment, respectively.

As shown in Table 1, glucan loss was only 10 % even though sample was disk refined prior to p-TsOH hydrolysis. Therefore, effect of pretreatments on degradation of cellulose was not discussed in this part. The chemical composition and yields of water insoluble solids were listed in Table 2. The hydrolysis temperature and acid concentration could also result in the changes of remaining rate of xylan and lignin (Figure 5). Compared to raw material, disk refined poplar was more vulnerable to *p*-TsOH hydrolysis due to the wood size reduction. Xylan, with the most hemicellulose in poplar, degraded with the increasing temperature and acid concentration. It was obviously seen that temperatures between 75 °C and 80 °C made little difference on R_xylan_ when the acid concentration was up to 80 wt %. Results in Table 2 and Figure 5 revealed that *p*-TsOH removed a substantial amount of lignin from poplar wood, especially in disk refined fibers. The minimum R_lignin_ in these experiments was obtained from disk refined poplar fiber using acid hydrolysis condition at P80T80t20. For this point of view, *p*-TsOH treatment was helpful to remove xylan and lignin but did not induce cellulose depolymerization.

### 3.5. Chemical Structure of Raw Material and Pretreated Poplar Wood

**Figure 6:**
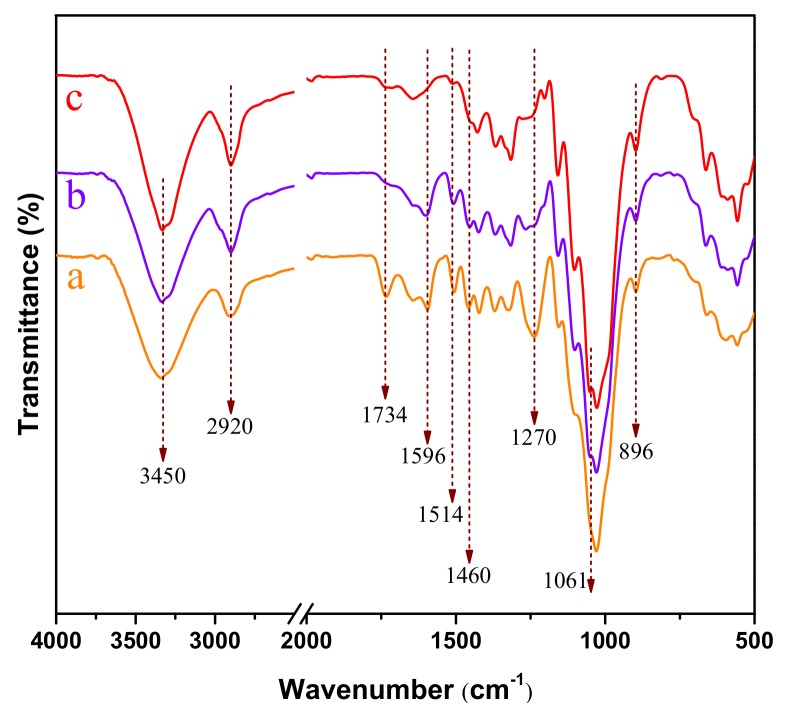
FTIR spectra of poplar wood under different treatment conditions. (**a**) Raw material; (**b**) P (**c**) RP.

The chemical compositions of raw material and corresponding treated poplar were investigated by FTIR in Figure 6. Because hot water extraction in this study mainly removed a portion of hemicellulose (xylan and mannan), therefore, spectra in raw poplar were similar to that in hydrolyzed sample. Herein, we compared the FTIR spectra of raw material, P and RP samples. The chemical bands were assigned and summarized in Table S1. The dominant peaks of OH-stretching and CH-stretching at 3450 cm^−1^ and 2920 cm^−1^ were presented in the entire spectra [26]. The peaks at 896 cm^−1^ and 1061 cm^−1^ were associated with C-H deformation vibrations and C-O-C stretching vibrations, respectively, these data showed typical cellulose structural characteristics as reported in many previous studies [27,28]. The band at 1460 cm^−1^, 1514 cm^−1^ and 1596 cm^−1^ were assign to C-H deformation vibration, aromatic skeletal vibration and C=C benzene ring vibration of lignin, respectively [29,30]. These peaks were observed in curve “b” after p-TsOH hydrolysis, suggesting that some part of lignin still existed in p-TsOH treated poplar, which was consistent with the results in Table 1. It was also proved that poplar size reduction by disk refining made lignin easier to remove. Interestingly, no difference was found between the spectra of cellulose structure in each sample. This result suggested that physical (disk refining) or chemical (p-TsOH hydrolysis) treatment did not alter the molecular structures of cellulose.

## 4. Conclusions

In this work, autohydrolysis and disk refining pretreatments were employed for the dissolution and fractionation of poplar wood. Autohydrolysis was a mild and green condition for hemicellulose dissolution. However, poplar wood with or without autohydrolysis had almost the same chemical composition after *p*-TsOH hydrolysis at a short time and low temperature. When using disk refining with low input prior to pure *p*-TsOH treatment, the removal of lignin and hemicellulose was enhanced. The delignification was especially doubled after mechanical and chemical treatments; however, the cellulose loss was minimal. As a result, the crystallinity of treated poplar was increased and chemical structure was changed after physicochemical treatment. Further work will be needed to remove the residual lignin to produce dissolving pulp from the fibers.

## Figures and Tables

**Table 1:** Chemical composition and crystallinity index of the poplar wood samples under different treatment conditions.

Sample Label ^1^	Glucan (%)	Xylan (%)	Mannan (%)	Lignin (%)	Solid Yield (%)	R_glucan_	R_xylan_	R_lignin_	CrI (%)
Raw material	46.53	15.38	4.46	23.73	100	1.00	1.00	1.00	60.6 ± 0.4
H	49.38	7.13	1.61	25.57	93.20	0.99	0.43	1.00	68.3 ± 0.4
P	60.44	7.27	2.83	18.55	76.61	0.99	0.36	0.60	69.9 ± 0.2
HP	60.53	6.08	2.28	20.01	75.25	0.98	0.30	0.63	74.0 ± 1.1
RP	71.87	4.44	3.10	5.92	56.88	0.88	0.16	0.14	78.5 ± 0.6
HRP	73.42	4.18	3.03	5.25	54.23	0.86	0.15	0.12	78.4 ± 0.3

^1^ H was autohydrolysis; P was *p*-toluenesulfonic acid hydrolysis at reaction condition P80T80t20, where P, T and t represent *p*-toluenesulfonic acid loading in wt %, reaction temperature in °C and duration in min, respectively; R was disk refining.

**Table 2:** Chemical composition and yields of water insoluble solids under different hydrotropic fractionation.

Sample Label ^1^	Glucan (%)	Xylan (%)	Mannan (%)	Klason Lignin (%)	Solid Yield (%)
**P70T75t20**	57.08 ± 0.36	9.20 ± 0.16	4.01 ± 0.05	21.75 ± 0.15	80.04
**P70T80t20**	56.19 ± 0.09	9.34 ± 0.41	3.97 ± 0.04	20.16 ± 0.28	77.42
**P75T75t20**	56.40 ± 0.23	8.02 ± 0.17	3.41 ± 0.42	19.54 ± 0.31	78.01
**P75T80t20**	57.78 ± 0.23	7.62 ± 0.13	3.00 ± 0.08	19.10 ± 0.38	76.98
**P80T75t20**	59.43 ± 0.42	7.21 ± 0.11	2.54 ± 0.55	19.35 ± 0.19	77.03
**P80T80t20**	60.33 ± 0.16	7.15 ± 0.17	2.94 ± 0.16	18.69 ± 0.20	75.61
**R+P70T75t20**	58.28 ± 0.21	7.41 ± 0.25	4.55 ± 0.40	14.34 ± 0.48	70.12
**R+P70T80t20**	63.49 ± 0.35	6.97 ± 0.11	4.36 ± 0.24	9.45 ± 0.11	64.67
**R+P75T75t20**	63.83 ± 0.48	6.56 ± 0.46	3.64 ± 0.53	11.23 ± 0.29	65.67
**R+P75T80t20**	66.21 ± 0.26	5.39 ± 0.35	3.89 ± 0.18	7.91 ± 0.04	62.06
**R+P80T75t20**	69.06 ± 0.32	4.82 ± 0.16	3.94 ± 0.11	8.47 ± 0.29	60.48
**R+P80T80t20**	71.95 ± 0.11	4.36 ± 0.12	3.47 ± 0.52	5.47 ± 0.64	56.88

^1^ P, T and t represent *p*-toluenesulfonic acid loading in wt %, reaction temperature in °C and duration in min, respectively; R was disk refining.

## Supplementary Materials

The following are available online at https://www.mdpi.com/2073-4360/11/4/685/s1, Table S1. List of chemical bands of lignocellulose.
