# Supplementary material for: Enhancement of Hydrotropic Fractionation of Poplar Wood Using Autohydrolysis and Disk Refining Pretreatment: Morphology and Overall Chemical Characterization
Source: Polymers (Basel). 2019 Apr 15;11(4):685. doi: 10.3390/polym11040685 (PMC6523484; doi:10.3390/polym11040685)
Supplement: Supplementary file 1 [file polymers-11-00685-s001.pdf]

Electronic Supplementary Information

Enhancement of Hydrotropic Fractionation of Poplar Wood using Autohydrolysis and Disk Refining Pretreatment: Morphology and Overall Chemical Characterization

Yanting Gu [1,2,*], Huiyang Bian [1,3], Liqing Wei [4] and Ruibin Wang [5]

[1] Jiangsu Co-Innovation Center of Efficient Processing and Utilization of Forest Resources, Nanjing Forestry University, Nanjing 210037, China; guyanting@njfu.edu.cn (Y.G.); hybian1992@njfu.edu.cn (H.B.)
[2] College of Furnishings and Industrial Design, Nanjing Forestry University, Nanjing 210037, China
[3] Jiang Provincial Key Lab of Pulp and Paper Science and Technology, Nanjing Forestry University, Nanjing 210037, China
[4] Forest Products Laboratory, U.S. Forest Service, U.S. Department of Agriculture, Madison, WI 53726, USA; liqingwei1325@gmail.com (L.W.)
[5] School of Materials and Energy, Center of Emerging Material and Technology, Guangdong University of Technology, Guangzhou 510006, China; wang.rb@gdut.edu.cn (R.W.)

* Correspondence: guyanting@njfu.edu.cn (Y.G.); Tel.: 86-25-85427528 (Y.G.)

Table S1. List of chemical bands of lignocellulose

Wavenumber (cm^{-1})	Band assignment
3420	O-H stretching in aliphatic and phenolic -OH
2920	C-H stretching (CH_3 and CH_2)
2937, 2841	C-H stretching in methyl and methylene groups
1734	C=O stretching (hemicellulose or lignin)
1705	C=O stretching in unconjugated ketones and carboxyl groups; aryl and α,β unsaturated esters
1596	C=C benzene ring vibration
1508	Aromatic skeletal vibration
1463	C-H asymmetric deformations in methyl and methylene groups
1423	Aromatic skeletal vibrations
1270	G ring breathing and C-O stretching
1216	C-O stretching in phenols and ethers
1113	Aromatic C-H in-plane deformations in S units
1033	Aromatic C-H in-plane deformations in G units; C-O deformations in primary alcohols
913	Aromatic C-H out-of-plane deformation (only in GS lignin type)
896	C-H deformation vibration
840	Aromatic C-H out-of-plane deformation in G units
